# Novel *RPE65* mutations associated with Leber congenital amaurosis in Chinese patients

**Published:** 2012-03-28

**Authors:** Fei Xu, Qiang Dong, Liang Liu, Hui Li, Xiaofang Liang, Ruxin Jiang, Ruifang Sui, Fangtian Dong

**Affiliations:** 1 Department of Ophthalmology, Peking Union Medical College Hospital, Peking Union Medical College and Chinese Academy of Medical Sciences, Beijing, China; 2 Taishan Medical College, Beijing, China; 3 Ocular Genetic Laboratory, Peking Union Medical College Hospital, Peking Union Medical College and Chinese Academy of Medical Sciences, Beijing, China

## Abstract

**Purpose:**

Retinal pigment epithelium-specific protein 65 kDa (*RPE65*) plays an essential role in vitamin A metabolism necessary for synthesizing the visual pigment 11-cis-retinal chromophore. Mutations in *RPE65* cause the childhood blindness disorder known as Leber congenital amaurosis (LCA), as well as autosomal recessive retinitis pigmentosa (RP). The purpose of this study was to identify *RPE65* mutations in Chinese patients with LCA, determine the prevalence of *RPE65* mutations in this cohort, and assess the clinical features of those patients with *RPE65* mutations.

**Methods:**

Detailed ocular examinations were performed, and genomic DNA was isolated with standard methods for genetic diagnosis. All 14 exons of *RPE65* were amplified with PCR and screened for mutation with direct DNA sequencing. Two hundred unrelated healthy Chinese subjects were screened to exclude nonpathogenic polymorphisms. Multiple alignments of eight eukaryotic *RPE65* orthologs were performed.

**Results:**

A total of 101 LCA patients, drawn from 100 unrelated families, were selected for mutation screening in the *RPE65* gene. Compound heterozygous missense mutations Leu67Arg and Tyr368Cys were identified in two affected sisters and segregated with their family. Four previously reported polymorphisms were identified in this study. No other disease-related mutation was detected. The frequency spectrum of variations in the *RPE6*5 gene was estimated to be 1% (1/100) in this cohort of Chinese patients with LCA. The two patients showed classical signs of LCA with relatively preserved central vision and retinal structure.

**Conclusions:**

The *RPE65* mutation is a rare cause of LCA in the Chinese population. Compound heterozygous missense mutations Leu67Arg and Tyr368Cys are related to a relatively mild LCA phenotype. Genetic characterization of patients with *RPE65* mutations is important for future rational therapies.

## Introduction

Leber congenital amaurosis (LCA, OMIM 204000) represents a severe form of inherited retinal dystrophy that accounts for 5% of all inherited retinopathies [1]. LCA is generally inherited in an autosomal recessive manner although some autosomal dominant families have been reported [2-4]. LCA is characterized by congenital blindness, nystagmus, and severely reduced or nondetectable electroretinogram (ERG) [1,5]. In addition, affected children sometimes have oculodigital sign, which refers to repetitive pressing, poking and rubbing their eyes with fingers or fists. However, certain phenotypic variations have also been identified in patients with LCA, including heterogeneity in retinal appearance, refractive errors, and photosensitivity [6]. Clinical heterogeneity may reflect the genetic heterogeneity of this retinal disorder.

LCA has been associated with mutations in 17 genes (RetNet). These genes have been discovered by various methods, including linkage analysis, the candidate gene approach, and homozygosity mapping. The identified functions of the proteins encoded by these genes are remarkably heterogeneous, including participation in the phototransduction cascade, structure of the photoreceptors, the retinoid cycle, RNA splicing, and so on [7]. Among the known disease genes, missense mutations in retinal pigment epithelium-specific protein 65 kDa (*RPE65*) were identified in a patient with LCA type II using the candidate gene approach [8]. The *RPE65* gene contains 14 coding exons spanning 20 kb and encodes the isomerase enzyme necessary for converting all-trans-retinol to 11-cis-retinal in the visual cycle [9]. According to published reports, the prevalence of *RPE65* mutations ranges from 1.7% to 16% in LCA cohorts from various geographical origins, with higher frequencies reported in the United States [10-12].

To date, more than 80 LCA-associated *RPE65* mutations have been identified (HGMD). Most of the previous genetic studies on LCA were performed in the Western population, and only limited data are available from Chinese patients [13]. The purpose of this study is to analyze the *RPE65* mutations in a cohort of Chinese patients with LCA and to describe detailed clinical features of patients with LCA with *RPE65* mutations.

## Methods

### Recruitment of subjects

All participants were identified at the Ophthalmic Genetics Clinic at Peking Union Medical College Hospital (PUMCH), Beijing, China. LCA was defined as severe visual impairment within the first year after birth, nystagmus, oculodigital sign, and severely reduced or nondetectable ERG. Family members of the probands were invited for a clinical and genetic assessment. Written informed consent was obtained either from the participating individuals or their guardians. This study was approved by the Institutional Review Board of PUMCH and adhered to the tenets of the Declaration of Helsinki and the Guidance on Sample Collection of Human Genetic Diseases by the Ministry of Public Health of China.

### Clinical evaluations

A full medical and family history was taken and ophthalmological examination performed. Each patient underwent standard ophthalmic examination: best corrected visual acuity according to Snellen charts, slit-lamp biomicroscopy, dilated indirect ophthalmoscopy, fundus photography if possible, and visual field tests. The retinal structure was examined with optical coherence tomography (OCT; Topcon, Tokyo, Japan). ERGs were performed (RetiPort ERG system; Roland Consult, Wiesbaden, Germany) using corneal “ERGjet” contact lens electrodes. The ERG protocol complied with the standards published by the International Society for Clinical Electrophysiology of Vision (ISCEV).

### Genetic studies

Genomic DNA was isolated from peripheral leukocytes using a QIAamp DNA Blood Midi Kit (Qiagen, Hilden, Germany) according to the manufacturer’s protocol. All 14 coding exons, including intron-exon boundaries of the *RPE65* gene, were amplified with PCR using primers published previously [10]. After purification, amplicons were sequenced using forward and reverse primers on an ABI 3730 Genetic Analyzer (ABI, Foster City, CA). Sequences were assembled and analyzed using Lasergene SeqMan software (DNASTAR, Madison, WI). The results were compared with the *RPE65* reference sequence. Direct sequencing was used to investigate the presence of a novel variation in 200 unrelated healthy Chinese control subjects to exclude nonpathogenic polymorphisms. Cosegregation analysis was performed in available family members. Multiple sequence alignment of eight eukaryotic *RPE65* homologs was performed and analyzed with an online analysis tool (HomoloGene). We also assessed the potential functional consequences of nucleotide changes using the online bioinformatics tool PolyPhen.

## Results

### Patient demographics

One hundred and one patients in 100 unrelated families with a clinical diagnosis of LCA were recruited for the study. All patients were of Chinese ethnicity. Only one patient was born of a consanguineous marriage.

### Mutation analysis

**Figure 1:**
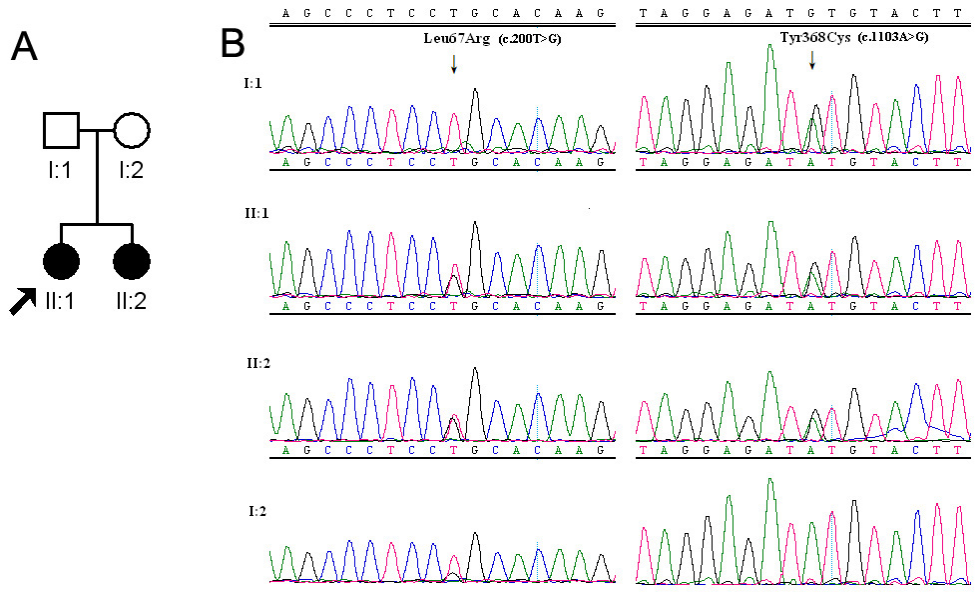
Sequence analysis of the *RPE65* gene missense mutation Leu67Arg (c.200T>G) and Tyr368Cys (c.1103A>G) in a family with two affected patients. **A**: A Chinese family with Leber congenital amaurosis (LCA) harbors mutations in the retinal pigment epithelium-specific protein 65 kDa (*RPE65*) gene. Squares indicate men; circles, women; black symbols, affected individuals; and the arrow indicates the proband. **B**: The figure shows sequencing results of the mutations in this family.

**Figure 2:**
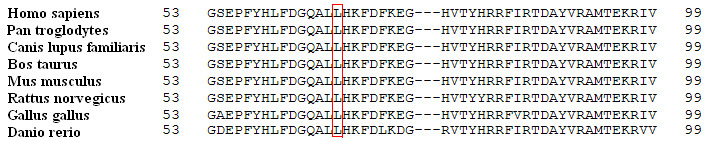
The figure shows multiple sequences alignment for genetic variants of the retinal pigment epithelium-specific protein 65 kDa (*RPE65*) gene in different species. The amino acids boxed in red indicate the position of Leu67Arg in the present study. This mutation occurs in highly conserved region.

Sequence analysis of the *RPE65* gene identified one novel missense mutation Leu67Arg (c.200T>G) and one previously reported missense mutation Tyr368Cys (c.1103A>G) in a family with two affected patients (Figure 1) [14]. The two affected sisters carried the mutations in compound heterozygous form. The mother carried the Leu67Arg mutation on one *RPE65* allele, and the father carried the Tyr368Cys mutation on one *RPE65* allele. The novel Leu67Arg mutation identified in this study was absent in 200 unrelated healthy Chinese control subjects and is highly conserved through the species, strongly indicating the pathogenicity of this novel missense variant (Figure 2). The novel variant was predicted to be “probably damaging” by PolyPhen. Four previously reported polymorphisms were identified in this study (Table 1, SNP).

**Table 1:** Polymorphisms detected in 100 unrelated LCA patients

**ID**	**Chr:bp**	**Alleles**	**HGVS names**	**Type**	**Amino acid**	**Number of patients**
rs2274321	1:68906514	G/A	ENST00000262340.5: c.643+22C>T	Intronic	-	3
rs12564647	1:68896945	T/G	ENST00000262340.5: c.1338+20A>C	Intronic	-	27
rs12145904	1:68903942	C/T	ENST00000262340.5: c.1056G>A	Synonymous coding	E352E	26
rs10626313	1:68894733–68894732	-/CT	ENST00000262340.5:c.*726_*727insAG	3 prime UTR	-	9

SNP website used.

### Clinical assessment

#### The 13-year-old proband (II:1)

**Figure 3:**
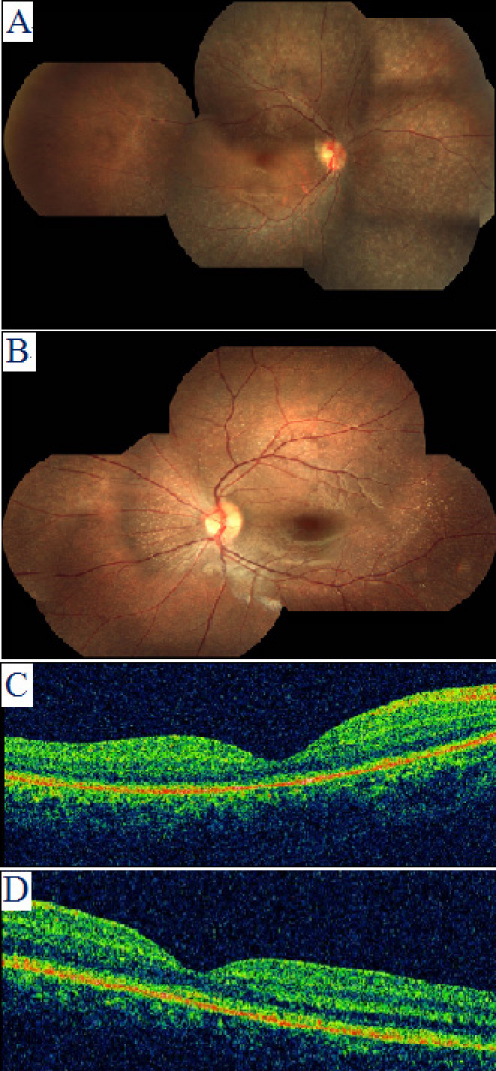
Fundus photograph and optical coherence tomography pictures of the two patients with retinal pigment epithelium-specific protein 65 kDa (*RPE65*) mutations. **A** and **C**: patient II:1; **B** and **D**: patient II:2.

**Figure 4:**
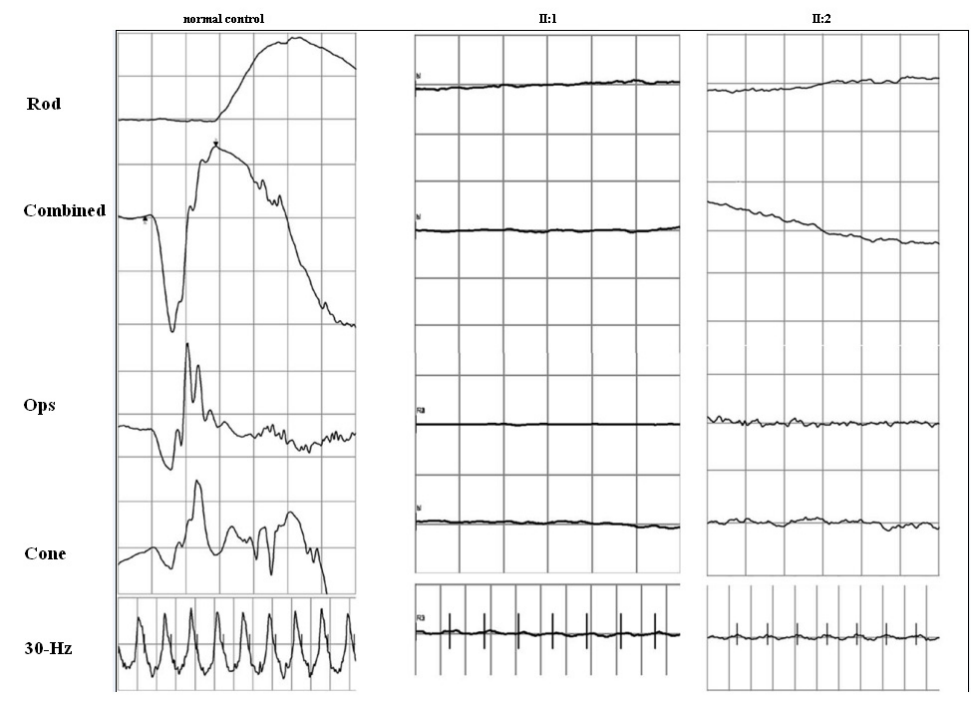
The figure depicts the electrophysiological changes of the two Chinese Leber congenital amarousis (LCA) patients. All recording conditions refer to International Society for Clinical Electrophysiology of Vision (ISCEV).

This affected girl had received a clinical diagnosis of retinitis pigmentosa at the age of 9 years and was referred to PUMCH for further evaluation. Her visual function was poor, and visual acuity was markedly decreased in dimmer conditions. Her best corrected visual acuity (BCVA) was 20/100 (OD) and 20/200 (OS) at the age of 9 years. The refractions were +2.50DS-3.00DC×165° (OD) and +3.00DC×105° (OS). Intermittent nystagmus was present. Color fundus montages showed mildly attenuated retinal vessels, normal foveal reflexes, and numerous grayish deposits in the midperipheral retina (Figure 3A). ERG was found to be almost unrecordable (Figure 4). When the proband was examined at her present age of 13, her BCVA was 20/100 in the right eye and 20/80 in the left eye. Her binocular vision reached 20/60. Nystagmus was noted. OCT showed an almost normal retinal microstructure with a detectable but thinned photoreceptor layer (Figure 3C).

#### The 7-year-old affected sister (II:2)

This affected sister had a similar complaint of visual defect as her older sister. On examination, her visual acuity was 20/80 (OU). The refractions were +7.00DS+1.00DC×80° (OD) and +6.50DS+1.00DC×120° (OS). Her visual acuity increased to 20/60 (OU) during the two-year follow-up. Mild nystagmus was observed. The anterior segment was normal. Fundus examination showed minimal attenuation of retinal vessels and many whitish punctuate lesions in the midperipheral retina (Figure 3B). An extensively thinned photoreceptor layer was documented with an OCT scan (Figure 3D). ERG recordings showed extinguished rod responses and severely diminished but recordable cone responses (Figure 4).

## Discussion

The frequencies of mutations in the *RPE65* gene vary remarkably in different populations. With the study presented here, we report one novel and one previously reported mutation in the Chinese cohort. Our study reveals a prevalence of 1% (1/100) in LCA cases due to *RPE65* mutations. This finding is similar to the findings in studies from India (1.7%) and Saudi Arabia (2.7%), but lower than in another study from China (5.7%) [13]. A possible reason for this difference could be the different enrollment criteria. As mutation in the *RPE65* gene may be related to LCA, severe early childhood onset retinal dystrophy, or even autosomal-dominant retinitis pigmentosa [15,16], different enrollment criteria may generate different positive results. However, according to previous studies, the variant frequency for the *RPE65* gene was no more than 6% in Asian patients with LCA, indicating that *RPE65*-associated LCA is rare in Asia.

Previous studies have demonstrated that mutations in *RPE65* may cause profound visual impairment at birth, and transient improvement with useful vision persisting up to the second decade [17-19]. A similar result was obtained in the current study: the proband had poor visual acuity in the first decade but showed mild improvement in BCVA when she was examined at the age of 13. The improvement in the visual function in the proband and her younger sister may be interpreted as pointing to epigenetic regulatory mechanisms induced by hormonal changes during puberty [20].

The two patients demonstrated normal appearance of the central retina; furthermore, relatively preserved central retinal architecture was shown with OCT. According to a study conducted by Dr. Paunescu [18], the funduscopic appearance of patients with *RPE65* mutations was normal or showed minor changes in the first decade. From the second decade on, most patients had progressive macular and/or peripheral changes. Our result was consistent with this study and further verified the theory that patients with *RPE65* mutations have better visual functions than typically seen in LCA. However, the retinal experience in these two patients was not exactly the same. The proband’s younger sister showed evidence of fine white retinal dots. This phenotype is not frequently observed in patients with *RPE65*-associated LCA. The white retinal dots may represent abnormal accumulations of retinyl esters, as has been demonstrated in animal models [21,22].

To date, multiple missense mutations in the *RPE65* gene have been identified in patients with inherited retinal dystrophies, and a wide range of disease severity has been associated with *RPE65* mutations, from congenital blindness LCA to adult-onset retinitis pigmentosa. With these initial reports, a possible link between the severity of the disease and the type of mutations in the *RPE65* gene remains to be elucidated [23-25]. RPE65 is an abundant membrane-associated protein in the retinal pigment epithelium, and this characteristic membrane association is essential for isomerohydrolase activity. Previous studies have shown that some missense mutations may lead to the instability as well as the mislocalization of RPE65, thus severely impairing its catalytic activity [26,27]. However, different missense mutations at the same residual may have different levels of influence on enzymatic activity [28]. The exact impacts of the two point mutations identified in the current study on the structure and function of RPE65 remain to be discovered.

Until recently, almost all types of inherited retinal degenerations, such as LCA, were considered incurable. However, gene therapy has lit a candle in the dark. In 2008, three human gene therapy clinical trials were reported involving a subretinal injection of adeno-associated virus vector delivering a wild-type copy of *RPE65*. Most patients exhibited some improvement in visual function, and there were no obvious adverse events [29-31]. Identifying patients with mutations in the *RPE65* gene has attained greater significance now that gene replacement trials have begun.
